# Direct, indirect, and reciprocal associations between perfectionism, compulsive exercise and eating disorder pathology in adolescents with eating disorders

**DOI:** 10.1007/s40519-024-01650-y

**Published:** 2024-03-24

**Authors:** Vinola Adams, Hunna J. Watson, Trevor Mazzucchelli, Emily Jones, Thomas Callaghan, Elizabeth Bills, Sarah J. Egan

**Affiliations:** 1 Discipline of Psychology, School of Population Health, Curtin University, Perth, Australia; 2 Department of Psychiatry, School of Medicine, University of North Carolina at Chapel Hill, Chapel Hill, USA; 3 Division of Paediatrics, School of Medicine, University of Western Australia, Perth, Australia; 4 enAble Institute, Faculty of Health Sciences, Curtin University, GPO Box U1987, Perth, WA 6847 Australia; 5 Eating Disorders Program, Child and Adolescent Health Service, Perth Children’s Hospital, Perth, Australia

**Keywords:** Perfectionism, Eating disorder, Compulsive exercise, Adolescents

## Abstract

**Background:**

There is a strong association between perfectionism and eating disorders. In a cognitive–behavioural model of compulsive exercise it has been suggested there are reciprocal associations between perfectionism, eating disorder pathology, and compulsive exercise. No study has examined if there is an indirect association between perfectionism and compulsive exercise through eating disorder pathology, which would inform a preliminary understanding of the cognitive–behavioural model of compulsive exercise.

**Methods:**

The sample included 301 adolescent females diagnosed with eating disorders (age *M* = 14.89, SD = 0.85, range 13–17). We tested models of direct and indirect associations of compulsive exercise in the relationship between perfectionism and eating disorder pathology, and direct and indirect associations of eating disorder pathology in the relationship between compulsive exercise and perfectionism.

**Results:**

Perfectionism was directly associated with eating disorder pathology and compulsive exercise. Perfectionism was indirectly associated with eating disorder pathology through compulsive exercise. Perfectionism also had an indirect association with compulsive exercise through eating disorder pathology.

**Discussion:**

The findings suggest it would be useful for future prospective research to examine the cognitive–behavioural model of compulsive exercise in adolescents with eating disorders. Compulsive exercise and perfectionism may be useful targets for future research to improve eating disorder treatment.

*Level of evidence* Level V: Opinions of respected authorities, based on descriptive studies, narrative reviews, clinical experience, or reports of expert committees.

## Introduction

Given the prevalence, peak onset, and adverse physical and mental health consequences of eating disorders in adolescence [30], it is important to understand constructs, including exercise, associated with eating disorder symptoms in youth. Compulsive exercise, characterised by an extreme and rigid urge to exercise, is consistently linked to eating disorders and implicated in clinical models of eating disorders [26]. Compulsive exercise can be a symptom of bulimia nervosa; however, it is also present across a range of eating disorder diagnoses [16]. Hence, there is a need to understand the role of compulsive exercise from a transdiagnostic perspective, across a broader range of eating disorder psychopathology.

In individuals with eating disorders, compulsive exercise is associated with greater risk of relapse, longer hospital admissions, suicidal behaviour, and premature treatment dropout [10, 26]. Harris et al. [16] proposed compulsive exercise as a transdiagnostic process in eating disorders, based on its association with adverse outcomes for recovery, including length of hospitalisation, greater relapse, and chronic outcomes. Compulsive exercise tends to be among the last eating disorder symptoms to subside [15]. It is important to examine compulsive exercise in adolescents with eating disorders given this is a developmental stage when exercise and eating-related attitudes develop [18].

Perfectionism is another transdiagnostic process associated with eating disorder symptoms [1, 33]. Taranis and Meyer [34] theorised that perfectionism is a maintaining factor of compulsive exercise in eating disorders. Fairburn et al.’s [9] widely cited transdiagnostic cognitive–behavioural theory of eating disorders suggests that clinical perfectionism is one of the four core maintaining mechanisms of an eating disorder, where an individual displays perfectionism in eating, shape, weight, and their control. This is supported by evidence that perfectionism is a transdiagnostic process which is both a risk and maintaining factor across eating disorders [6].

**Fig. 1:**
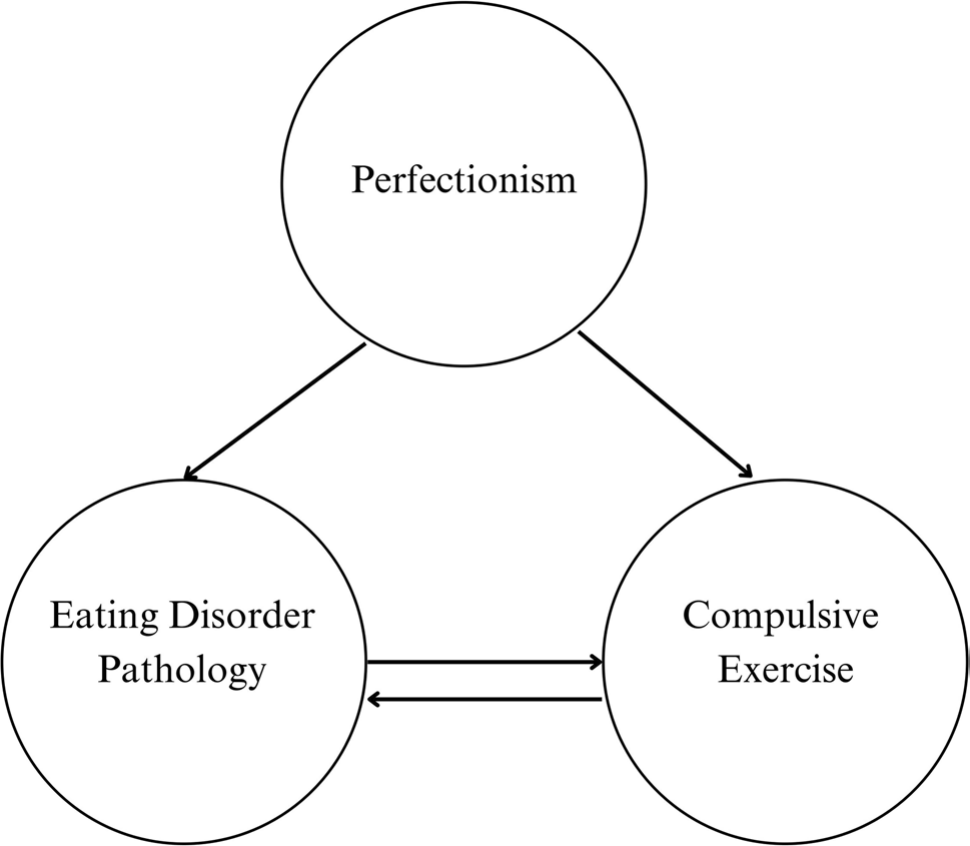
Theorised paths between perfectionism, eating disorder pathology, and compulsive exercise in Meyer et al.’s [26] cognitive–behavioural theory of compulsive exercise

Perfectionism has been found to predict higher eating disorder symptoms and lower remission in adolescents with eating disorders [19]. Meta-analyses show a consistent association between perfectionism and eating disorder symptoms in adolescents [1, 24]. In a cognitive–behavioural model of compulsive exercise developed by Meyer et al. [26], perfectionism is proposed as a direct maintaining factor of both compulsive exercise and eating disorder pathology [26] (see Fig. 1). Perfectionism is thought to maintain eating disorder pathology indirectly through compulsive exercise [26]. For example, an individual may apply perfectionistic standards to their exercise, i.e., ‘I must always exercise every day for at least 1 h, as hard as possible’, which reinforces both compulsive exercise and eating disorder symptoms.

Meyer et al.’s [26] systematic review summarised literature reporting that perfectionism and eating disorder pathology were correlated with compulsive exercise in young adults with eating disorders. The cognitive–behavioural model of compulsive exercise [26] has been examined in research demonstrating perfectionism is directly and indirectly associated with eating disorder pathology through compulsive exercise in both a non-clinical adult sample [4], and adolescents with eating disorders who were underweight [3].

In addition to Meyer et al.’s [26] predictions that compulsive exercise and eating disorder pathology reciprocally reinforce each other, a reciprocal relation was also proposed where perfectionism is theorised to reinforce compulsive exercise, indirectly through eating disorder pathology [26]. To examine the model [26] in an adolescent clinical population, it would be helpful to examine the reciprocal association proposed in the second pathway in the model. Specifically, it would be useful to examine the association between perfectionism and compulsive exercise and whether eating disorder pathology mediates this association [4].

Compulsive exercise and perfectionism have been examined to date only in an underweight clinical sample of adolescents with eating disorders [3]. It would be helpful to examine these constructs in relation to eating disorder symptoms in a broader clinical adolescent eating disorder population, not only an underweight sample. The transdiagnostic model of eating disorders [9] and cognitive–behaviour therapy-enhanced (CBT-E [7]), is based on the premise that individuals with eating disorders frequently ‘migrate’ between diagnostic categories. There is a high degree of overlap between eating disorder diagnoses [7, 9], including in adolescents [23]. In the current study, we framed the research based on a transdiagnostic approach. We were interested in the relationships between perfectionism, compulsive exercise and eating disorders across a broad range of adolescents, rather than differences between specific diagnostic groups. A transdiagnostic approach may have utility in informing future research directions in clinical settings, including the eating disorder program from which participants were included for this study, which is a transdiagnostic rather than specific disorder-based treatment program.

Understanding the cognitive behavioural model of compulsive exercise Meyer et al. [26] may also assist in informing if future prospective research should focus on confirming the pathways predicted in the model and the utility of treatment research into compulsive exercise in the context of eating disorders. It is critical to examine constructs of relevance to adolescent eating disorders, such as compulsive exercise and perfectionism, to identify potentially novel targets in the future given current treatments for adolescents with eating disorders are less than ideal and yield remission rates of only ~ 40% [25].

The aim of the current study was to examine direct, indirect, and reciprocal associations between compulsive exercise, perfectionism, and eating disorder pathology, in adolescents with eating disorders. The overall purpose of the study was to provide a preliminary examination of components of Meyer et al.’s [26] model, within the constraints and limitations of a cross-sectional design, to inform whether it would be useful for future prospective research to examine the model in a longitudinal study. We did not control for eating disorder diagnosis, since we framed the study from a transdiagnostic approach [7, 9]. Hence, we did not restrict the sample to only underweight individuals as Creswell et al. (2022) did, to gain a broader understanding of the associations between variables across the eating disorder diagnoses participants met in the sample. Based on the cognitive–behavioural model of compulsive exercise [26], it was hypothesised that there would be a direct and indirect relationship between perfectionism and eating disorder pathology through compulsive exercise, and that there would be a direct and indirect relationship between perfectionism and compulsive exercise through eating disorder pathology.

## Methods

### Participants

The participants were part of the Helping to Outline Paediatric Eating (HOPE) Disorders project, an ongoing prospective registry database of children and adolescents receiving treatment for an eating disorder (see [38] for further details of the database). The sample consisted of 301 adolescent females (age *M* = 14.89, SD = 0.85, age range 13–17 years and 10 months) with eating disorders (see Table 1). Participants were eligible if they met the following inclusion criteria: (1) female gender, (2) age between 13 and 17 years and 11 months, (3) a diagnosis of an eating disorder according to the Diagnostic and Statistical Manual of Mental Disorders-5 (DSM-5; American Psychiatric Association [APA], 2013), and (4) caregiver and participant consent to participate. Exclusion criteria were not answering the questionnaires included in this study, hence missing data was excluded. Males were excluded since they comprise a very small percentage (less than 7%; [31]) of participants, to ensure the results were generalisable to female clinical adolescent samples.

**Table 1:** Demographic information and DSM-5 diagnoses of participants (*N* = 301)

Eating disorder information	*M*	SD	*N*	%
CDC BMI z-score	− 1.43	1.28	294	
History of presenting problem (months)	10.24	7.91	260	
Age of onset (years)	14.01	1.05	260	
Binge eating episodes within 1 month	2.59	9.75	291	
Purging episodes within 1 month				
Self-induced vomiting	9.24	17.23	297	
Laxative misuse	1.25	7.79	299	
Diuretic misuse	1.27	11.61	296	
Diagnosis				
AN-R			106	35.2
AN-B/P			21	7.0
Aty-AN (OSFED)			88	29.2
BN			26	8.6
BN (OSFED)			10	3.3
Purging			4	1.3
UFED			45	15.0
Exercise information				
Presence of exercise			191	63.5
Absence of exercise			98	32.6
Hours spent exercising within 1 month	43	77.63	282	

CDC BMI z-score = Center for Disease Control and Prevention body mass index; AN-R = Anorexia nervosa–restricting type; AN-B/P = Anorexia nervosa–binge-eating/purging type; Aty-AN (OSFED) = Atypical anorexia nervosa (other specified feeding or eating disorder); BN = Bulimia nervosa; BN (OSFED) = Bulimia nervosa (other specified feeding or eating disorder); Purging = Purging disorder; UFED = Unspecified feeding or eating disorder

Patients were engaged in the Eating Disorders Program, at the Perth Children’s Hospital, Western Australia (WA). The treatment program is multidisciplinary and spans inpatient and outpatient treatment, and is the only public statewide specialist treatment centre for eating disorders in children and adolescents in WA. Participants were assessed from April 2012 to December 2019 as part of the intake assessment conducted prior to treatment. Eating disorder diagnoses were based on carer and child interviews with the child adapted Eating Disorder Examination (EDE; [8]) and medical records [38]. The present study, unlike Creswell et al.’s (2022), did not have specific weight criteria for inclusion or exclusion, resulting in a sample that partially overlaps with Creswell et al.’s underweight clinical sample.

### Measures

#### BMI z-scores

Measurements of weight and height were gathered during the intake medical assessment, to calculate BMI z-scores based on Centers for Disease Control and Prevention growth charts [20].

### Perfectionism

Perfectionism was measured using the six-item perfectionism subscale of the Eating Disorders Inventory (EDI-P; [13]). Items are scored on a 6-point Likert scale, from 1 to 6, with higher scores indicating greater perfectionism. The EDI-P score is derived from the mean score of the six items. Previous literature has shown the EDI-P to have good reliability, discriminant, and criterion validity [2]. The subscale has good convergent validity with other perfectionism measures [21]. The EDI-P has demonstrated good internal consistency (e.g., [3]). In the current study, the EDI-P had acceptable internal consistency (α = 0.77).

### Compulsive exercise

The compulsive exercise test (CET; [35]) is a 24-item self-report measure designed to assess key characteristics of compulsive exercise. Taranis et al. [35] found their data to fit five subscales, *weight control exercise* (e.g., “I exercise to burn calories and lose weight”), *avoidance and rule-driven behaviour* (e.g., “I feel like I've let myself down if I cannot exercise”), *lack of exercise enjoyment* (e.g., “I find exercise a chore”), *exercise rigidity* (e.g., “My weekly exercise pattern is repetitive”), and *mood improvement* (e.g., “Exercise improves my mood”). The five-factor structure has been found in non-clinical samples of adolescents [14] and university students [35] but did not fit a sample of adolescents with eating disorders [10]. Given that in clinical populations of adolescents with eating disorders, a five-factor solution has not been found, it has been suggested that the CET total score should be used instead of subscales scores [10]. Goodwin et al. [14] found the CET total score to be a valid and reliable measure of compulsive exercise among adolescents, and Harris et al. [16] reported the CET total score is a valid measure of compulsive exercise in adults with anorexia nervosa. Hence, the CET total score was used as a measure of compulsive exercise, rather than subscale scores. The CET as a total score has previously shown high internal consistency (α = 0.85; [35]) and had excellent internal consistency in the current study (α = 0.92).

### Eating disorder pathology

Eating disorder pathology was measured using the child-adapted version of the Eating Disorder Examination (EDE; [8]), a semi-structured interview which has been adapted by the Eating Disorders Program at Perth Children’s Hospital. The EDE has a global score derived from four subscales: restraint, eating, shape, and weight eating concerns [8]. The factorial validity of the child-adapted EDE was supported in a clinical sample of children and adolescents with eating disorders [28]. The child-adapted EDE has demonstrated acceptable validity and reliability in clinical samples of children and adolescents with eating disorders [28]. In the current study, the EDE global had excellent internal consistency (α = 0.93).

### Procedure

Perth Children’s Hospital Human Research Ethics Committee (HREC) provided ethics approval for this project (approval number: RGS0000002596), with reciprocal approval for this project granted through Curtin University HREC (approval number: HRE 2021-0389). Data collection for the HOPE project was achieved through a routine intake assessment using clinical and research instruments completed by patients, parents, and clinicians [38]. The interview and self-report measures included in this study were completed by Clinical Psychologists working at the eating disorders program, who received training and supervision in the use of these instruments by a Senior Clinical Psychologist with extensive experience in treatment of eating disorders. Medical assessment variables were completed by specialist medical doctors in the team with extensive experience of treatment of eating disorders in adolescents. Informed consent was obtained from participants and their parents/carer to have their anonymous data from the HOPE registry used for appropriate research purposes.

### Statistical analysis

SPSS (Version 23) and R (Version 1.2.27.0) were used to analyse the data in this study. Following Cresswell et al. [3], path analyses with bootstrapping were conducted to estimate the direct, indirect and total effects, allowing for a thorough evaluation of the interrelationships between the study variables. Bootstrapping involves resampling the data to determine the significance of the indirect effects and obtain more accurate and reliable confidence intervals for mediation models. The Jamovi Medmod R package was used to test direct and indirect associations using parametric bootstrapping with 1000 draws of the data. The magnitude of the effect sizes for both the direct and indirect pathways were expected to be between small and medium. An a priori power analysis demonstrated that 162 participants were required to achieve an 80% chance of determining this association at an alpha level of 0.05, therefore, our sample size of 301 adolescents was adequate [11].

## Results

### Descriptive statistics and correlation analyses

Means, standard deviations, and correlations for the study variables are shown in Table 2. When testing for direct and indirect effects, there should be significant correlations between both the direct and indirect variables [37]. This requirement was met for both models tested, with significant correlations found between perfectionism, compulsive exercise, and eating disorder pathology.

**Table 2:** Descriptive statistics and correlations between perfectionism, compulsive exercise, and eating disorder pathology

Variable	1	2	3	4
1. Age	–			
2. Perfectionism	0.05	–		
3. Compulsive exercise	0.10	0.29**	–	
4. Eating disorder pathology	0.23**	0.34**	0.57**	–
Mean	14.89	3.83	2.30	3.70
Standard deviation	0.85	1.03	1.04	1.50

*N* = 301. **p < 0.01 (2-tailed)

### Association between perfectionism and eating disorder pathology, through compulsive exercise

**Fig. 2:**
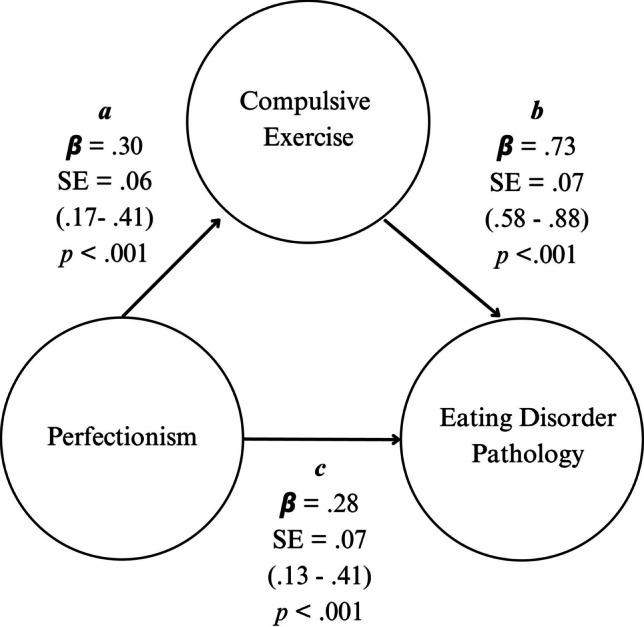
Model showing direct and indirect effects of perfectionism on eating disorder pathology through compulsive exercise, with standardised regression coefficients (β), standard error, and 95% confidence intervals for each path

Our first model examined the direct and indirect associations of perfectionism with eating disorder pathology through compulsive exercise. Perfectionism and compulsive exercise accounted for 35.4% of the total variance in eating disorder pathology, *R*^*2*^ = 0.35, adjusted *R*^*2*^ = 0.35, *F* (2, 298) = 81.68, *p* < 0.001, with a large effect size (*f*^2^ = 0.54; Cohen, 1988). Significant standardised regression coefficients were found for all paths, as shown in Fig. 2. Participants who reported higher perfectionism reported higher compulsive exercise (path *a*) = 0.296, 95% CI [0.173–0.407], *p* < 0.001. Those who reported higher compulsive exercise reported greater eating disorder pathology (path *b*) = 0.732, 95% CI [0.583–0.879], *p* < 0.001, and those with greater perfectionism reported higher eating disorder pathology (path *c*) = 0.277, 95% CI [0.133–0.413], *p* < 0.001. As indicated by bootstrapping, there was a significant indirect association between perfectionism and eating disorder pathology through compulsive exercise (path *ab*) = 0.217, 95% CI [0.127–0.313], *p* < 0.001. The total association (i.e., direct and indirect associations in combination) of perfectionism with eating disorder pathology was 0.494, 95% CI [0.336–0.652], *p* < 0.001. In summary, perfectionism was related to eating disorder pathology directly and indirectly through compulsive exercise.

### Association between perfectionism and compulsive exercise, through eating disorder pathology

**Fig. 3:**
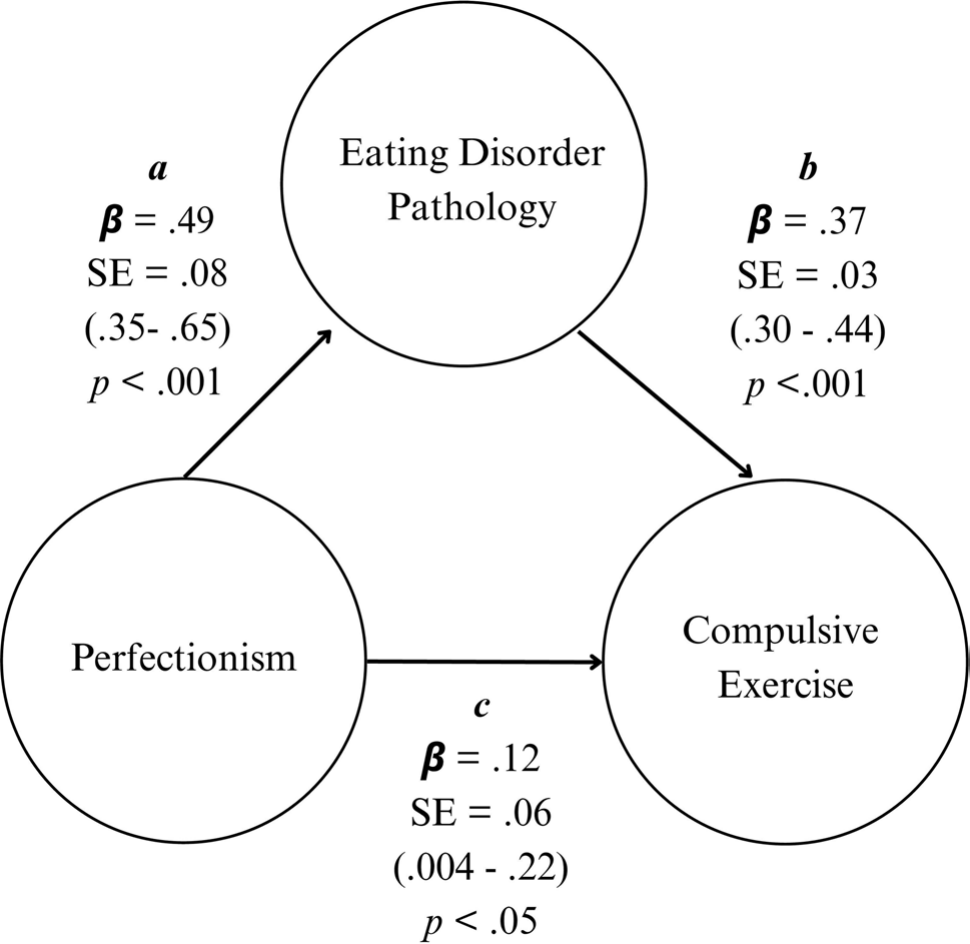
Model showing direct and indirect effects of perfectionism on compulsive exercise through eating disorder pathology, with standardised regression coefficients (β), standard error, and 95% confidence intervals for each path

Our second model examined direct and indirect effects of perfectionism on compulsive exercise through eating disorder pathology. Perfectionism and eating disorder pathology accounted for 33.2% of the variance in compulsive exercise, *R*^*2*^ = 0.33, adjusted *R*^*2*^ = 0.33, *F* (2, 298) = 74.12, *p* < 0.001, with a large effect size (*f*^2^ = 0.49; Cohen, 1988). Significant standardised regression coefficients were found for all paths (Fig. 3). Participants who reported higher perfectionism reported higher eating disorder pathology (path *a*) = 0.494, 95% CI [0.347–0.653], *p* < 0.001. Those who reported higher eating disorder pathology reported higher compulsive exercise (path *b*) = − 0.367, 95% CI [0.298–0.435], *p* < 0.001. Those who reported higher perfectionism reported higher compulsive exercise (path *c*) = 0.115, 95% CI [0.004–0.224], *p* < 0.05. As indicated by bootstrapping, there was a significant indirect effect between perfectionism and compulsive exercise through eating disorder pathology (path *ab*) = 0.181, 95% CI [0.120–0.251], *p* < 0.001. The total effect (i.e., direct and indirect effects in combination) of perfectionism on compulsive exercise was 0.296, 95% CI [0.176–0.410], *p* < 0.001. In summary, perfectionism was related to compulsive exercise directly and indirectly through eating disorder pathology.

## Discussion

In this cross-sectional study of female adolescents with eating disorders, perfectionism exhibited direct associations with eating disorder pathology and compulsive exercise. We also observed indirect associations, with eating disorder pathology mediated by compulsive exercise, and compulsive exercise mediated by eating disorder pathology.

The present study, the first of its kind in a broad clinical adolescent eating disorder population, contributes to the utility of future prospective, longitudinal research examining Meyer et al.’s [26] model. Prospective, longitudinal designs should be used to understand the theorised direct, indirect, and reciprocal paths linking perfectionism, eating disorder pathology, and compulsive exercise. Given the limits of cross-sectional mediation analysis not being able to infer longitudinal relations [29], we could not draw conclusions about whether the inferred reciprocal pathways in Meyer et al.’s [26] model were supported. However, our preliminary, cross-sectional findings are broadly consistent with the hypotheses of cognitive–behavioural theories of compulsive exercise [26] and eating disorders [9].

Our study extended Cresswell et al.’s [3] findings, by providing novel, albeit cross-sectional, insights into these associations, and by expanding beyond underweight clinical presentations. The results align with previous research and add to the growing body of literature that perfectionism is associated with both compulsive exercise and eating disorder pathology in adolescents (e.g., [1, 3]).

Future research should examine prospective, longitudinal relationships between perfectionism, compulsive exercise and eating disorder symptoms. Consistent with Meyer et al.’s [26] model, compulsive exercise may be one of the mechanisms through which perfectionism is related to eating disorder pathology. Several conceivable routes may underscore this connection, which would be useful to examine in future prospective studies. One hypothesis to explore is whether individuals with compulsive exercise perceive control over their exercise routines as a way to manage emotional distress. Future prospective research may also explore whether an intense focus on exercise may trigger body checking and social comparison and subsequent increased desire for thinness and body dissatisfaction.

The clinical implications of this line of research are ultimately to improve treatment outcomes for eating disorders. Building on Cresswell et al.’s [3] suggestion, considering the documented success of CBT for perfectionism (CBT-P) (e.g., see [5]) in reducing eating disorder symptoms in adolescents [12, 32], and compulsive exercise and eating disorder symptoms in adults [36], future research could examine the efficacy of CBT-P for compulsive exercise in adolescents with eating disorders.

Research in adults with anorexia nervosa indicates that treating compulsive exercise can lead to reduced eating disorder symptoms [16, 17]. Therefore, future research should also investigate the treatment of compulsive exercise for adolescents with eating disorders, specifically comparing the efficacy of CBT-P [5] to compuLsive Exercise Activity theraPy (LEAP; [17]), which incorporates psychoeducation based on Meyer et al.’s [26] model. This comparative analysis, as suggested by Harris et al. [16], could determine whether LEAP or CBT-P is superior. In addition, exploring whether CBT-P or LEAP could be beneficial as integrated components in standard treatment when compulsive exercise and perfectionism are elevated, based on an individualised case formulation, would be valuable.

### Strengths and limits

A strength was the large sample of adolescents diagnosed with eating disorders. Further, this study addresses the important variable of compulsive exercise in eating disorders, which has the potential to inform research on innovative treatment approaches. There were also numerous limitations. A significant limitation was the cross-sectional design did not allow causal inferences about the associations between perfectionism, compulsive exercise, and eating disorder symptoms. There are well known limits on conclusions that can be drawn from cross-sectional mediation (see O’Laughlin et al., [29] for further details), hence the results should be viewed tentatively and as preliminary and supporting the utility of future prospective research examining the constructs. Further, there was some overlap between the construct of compulsive exercise measured on the CET and items relating to drive exercise on the global score of the ChEDE used to assess eating disorder symptoms. Future research may consider for example measuring compulsive exercise in relation to eating disorder subscales which do not pertain to driven exercise. It would also be useful for future research to extend beyond mediation and consider examining whether perfectionism is a moderator of the association between compulsive exercise and eating disorder variables.

Although we framed the study from a transdiagnostic perspective [7, 9], most participants (71%) were diagnosed with anorexia nervosa and no participants with binge-eating disorder were included. While compulsive exercise is considered a transdiagnostic feature of eating disorders [27], research suggests the characteristics of compulsive exercise may vary across eating disorder diagnoses [22]. Hence, the generalisability of our results to a community-based population of adolescents with eating disorders is limited. Future research could examine differences between diagnostic groups on the associations between perfectionism, compulsive exercise and eating disorder symptoms, when there is a clinical sample where all eating disorder diagnoses can be compared, including binge-eating disorder. A further limitation was the female only sample. The inclusion of male and gender diverse samples in future research is required, as there may be a different pattern of association in other genders with the variables investigated.

## Conclusion

The results suggest that it is worthwhile for a future prospective study to examine the reciprocal pathways in Meyer et al.’s [26] cognitive–behavioural model of compulsive exercise. Future research should also examine treatment for perfectionism and compulsive exercise.

### What is already known on this subject?

Perfectionism and compulsive exercise are associated with eating disorders. Compulsive exercise has direct and indirect associations between perfectionism and eating disorder symptoms. A cognitive–behavioural model of compulsive exercise theorises reciprocal associations between eating disorder symptoms, compulsive exercise and perfectionism but this had not been tested.

### What this study adds?

There were reciprocal associations between eating disorder symptoms, perfectionism and compulsive exercise. The results support the utility of future prospective, longitudinal research examining the theorised reciprocal pathways in the cognitive–behavioural model of compulsive exercise.

## Data Availability

The data are available upon reasonable request and subject to institutional approvals.

## References

[1] Bills E, Greene D, Stackpole R, Egan SJ (2023). Perfectionism and eating disorders in children and adolescents: a systematic review and meta-analysis. Appetite.

[2] Clausen L, Rosenvinge J, Friborg O, Rokkedal K (2010). Validating the Eating Disorder Inventory-3 (EDI-3): a comparison between 561 female eating disorders patients and 878 females from the general population. J Psychopathol Behav Assess.

[3] Cresswell C, Watson HJ, Jones E, Howell JA, Egan SJ (2022). The role of compulsive exercise in the relationship between perfectionism and eating disorder symptoms in underweight adolescents with eating disorders. Eat Behav.

[4] Egan S, Bodill K, Watson H, Valentine E, Shu C, Hagger M (2017). Compulsive exercise as a mediator between clinical perfectionism and eating pathology. Eat Behav.

[5] Egan SJ, Wade TD, Shafran R, Antony MM (2014). Cognitive-behavioral treatment of perfectionism.

[6] Egan S, Wade T, Shafran R (2011). Perfectionism as a transdiagnostic process: a clinical review. Clin Psychol Rev.

[7] Fairburn C (2008). Cognitive behaviour therapy and eating disorders.

[8] Fairburn C, Cooper Z (1993). The eating disorder examination.

[9] Fairburn C, Cooper Z, Shafran R (2003). Cognitive behaviour therapy for eating disorders: a “transdiagnostic” theory and treatment. Behav Res Ther.

[10] Formby P, Watson H, Hilyard A, Martin K, Egan S (2014). Psychometric properties of the Compulsive Exercise Test in an adolescent eating disorder population. Eat Behav.

[11] Fritz MS, MacKinnon DP (2007). Required sample size to detect the mediated effect. Psychol Sci.

[12] Galloway R, Watson HJ, Greene D, Shafran R, Egan SJ (2022). The efficacy of randomised controlled trials of cognitive behaviour therapy for perfectionism: a systematic review and meta-analysis. Cogn Behav Ther.

[13] Garner DM (2004) EDI 3: Eating Disorder Inventory-3: Professional manual. Odessa: Psychological Assessment Resources

[14] Goodwin H, Haycraft E, Taranis L, Meyer C (2011). Psychometric evaluation of the compulsive exercise test (CET) in an adolescent population: links with eating psychopathology. Eur Eat Disord Rev.

[15] Hallward L, Duncan LR (2021). Compulsive exercise is a socially acceptable prison cell: exploring experiences with compulsive exercise across social media. Int J Eat Disord.

[16] Harris A, Mannon H, Hay P, Aouad P, Arcelus J, Attia E, Crosby R, Madden S, Meyer C, Touyz S (2024). Assessment and treatment of compulsive exercise in anorexia nervosa: a combined investigation of Compulsive Exercise Activity Therapy (LEAP) and Compulsive Exercise Test subscales. Eat Behav.

[17] Hay P, Touyz S, Arcelus J, Pike K, Attia E, Crosby RD, Madden S, Wales J, LaPuma M, Heriseanu AI, Young S, Meyer C (2018). A randomized controlled trial of the compulsive exercise activity therapy (LEAP): a new approach to compulsive exercise in anorexia nervosa. Int J Eat Disord.

[18] Jarosz E, Krug I, Letcher P, Olsson C (2014). A longitudinal study of disordered eating in Australian adolescents: modelling psychosocial and individual risk factors. J Eat Disord.

[19] Johnston J, Shu C, Hoiles K, Clarke P, Watson H, Dunlop P, Egan S (2018). Perfectionism is associated with higher eating disorder symptoms and lower remission in children and adolescents diagnosed with eating disorders. Eat Behav.

[20] Kuczmarski RJ, Ogden CL, Guo SS, Grummer-Strawn LM, Flegal KM, Mei Z (2002). 2000 CDC growth charts for the United States: methods and development. Vital Health Stat Ser 11 Data Natl Health Surv.

[21] Lampard A, Byrne S, McLean N, Fursland A (2012). The Eating Disorder Inventory-2 Perfectionism scale: factor structure and associations with dietary restraint and weight and shape concern in eating disorders. Eat Behav.

[22] Levallius J, Collin C, Birgegard A (2017). Now you see it, now you don't: compulsive exercise in adolescents with an eating disorder. J Eat Disord.

[23] Limburg K, Shu CY, Watson HJ, Hoiles KJ, Egan SJ (2018). Implications of DSM-5 for the diagnosis of paediatric eating disorders. Int J Eat Disord.

[24] Livet A, Navarri X, Pomerleau PP, Champagne S, Yunus FM, Chadi N, McVey G, Conrod P (2023). Perfectionism in children and adolescents with eating-related symptoms: a systematic review and a meta-analysis of effect estimates. Adolescents.

[25] Lock J, Le Grange D (2019). Family-based treatment: where are we and where should we be going to improve recovery in child and adolescent eating disorders. Int J Eat Disord.

[26] Meyer C, Taranis L, Goodwin H, Haycraft E (2011). Compulsive exercise and eating disorders. Eur Eat Disord Rev.

[27] Monell E, Levallius J, Forsén Mantilla E, Birgegård A (2018). Running on empty—a nationwide large-scale examination of compulsive exercise in eating disorders. J Eat Disord.

[28] O’Brien A, Watson H, Hoiles K, Egan S, Anderson R, Hamilton M, Shu C, McCormack J (2015). Eating disorder examination: factor structure and norms in a clinical female pediatric eating disorder sample. Int J Eat Disord.

[29] O’Laughlin KD, Martin MJ, Ferrer E (2018). Cross-sectional analysis of longitudinal mediation processes. Multivar Behav Res.

[30] Qian J, Wu Y, Liu F, Zhu Y, Jin H, Zhang H, Wan Y, Li C, Yu D (2022). An update on the prevalence of eating disorders in the general population: a systematic review and meta-analysis. Eat Weight Disord.

[31] Shu CY, Limburg K, Harris C, McCormack J, Hoiles KJ, Hamilton MJ, Watson HJ (2015). Clinical presentation of eating disorders in young males at a tertiary setting. J Eat Disord.

[32] Shu C, Watson HJ, Anderson RA, Wade TD, Kane RT, Egan SJ (2019). A randomized controlled trial of unguided internet cognitive behavior therapy for perfectionism: impact on risk for eating disorders. Behav Res Ther.

[33] Stackpole R, Greene D, Bills E, Egan SJ (2023). A systematic review and meta-analysis of the relationship between perfectionism and eating disorder symptoms in adults. Eat Behav.

[34] Taranis L, Meyer C (2010). Perfectionism and compulsive exercise among female exercisers: high personal standards or self-criticism?. Personality Individ Differ.

[35] Taranis L, Touyz S, Meyer C (2011). Disordered eating and exercise: development and preliminary validation of the compulsive exercise test (CET). Eur Eat Disord Rev.

[36] Valentine E, Bodill K, Watson HJ, Hagger MS, Kane RT, Anderson RA, Egan SJ (2018). A randomized controlled trial of unguided internet cognitive behavioral treatment for perfectionism in individuals who engage in regular exercise. Int J Eat Disord.

[37] Valeri L, VanderWeele TJ (2013). Mediation analysis allowing for exposure–mediator interactions and causal interpretation: theoretical assumptions and implementation with SAS and SPSS macros. Psychol Methods.

[38] Watson H, McCormack J, Hoiles K, Forbes D, Potts J (2013). The HOPE (Helping to Outline Paediatric Eating Disorders) Project: development and debut of a paediatric clinical eating disorder registry. J Eating Disord.

